# Rheological Properties of Weak Gel System Cross-Linked from Chromium Acetate and Polyacrylamide and Its Application in Enhanced Oil Recovery After Polymer Flooding for Heterogeneous Reservoir

**DOI:** 10.3390/gels10120784

**Published:** 2024-12-01

**Authors:** Yunqian Long, Chenkan Zhang, Dandan Yin, Tao Huang, Hailong Zhang, Ming Yue, Xiaohe Huang

**Affiliations:** 1 School of Petrochemical Engineering & Environment, Zhejiang Ocean University, Zhoushan 316022, China; zhangck2024@163.com (C.Z.); yindandan@zjou.edu.cn (D.Y.); huangtaozjou@126.com (T.H.); zhanghailong@zjou.edu.cn (H.Z.); 2 National & Local Joint Engineering Research Center of Harbor Oil & Gas Storage and Transportation Technology, Zhejiang Ocean University, Zhoushan 316022, China; 3 Zhejiang Key Laboratory of Petrochemical Environmental Pollution Control, Zhejiang Ocean University, Zhoushan 316022, China; 4 School of Civil and Resources Engineering, University of Science and Technology Beijing, Beijing 100083, China; yueming0101@163.com

**Keywords:** rheological property, weak gel system, conformance control, sweep efficiency, enhanced oil recovery

## Abstract

Long-term polymer flooding exacerbates reservoir heterogeneity, intensifying intra- and inter-layer conflicts, which makes it difficult to recover the remaining oil. Therefore, further improvement in oil recovery after polymer flooding is essential. In this study, a weak gel system was successfully synthesized, and possesses a distinct network structure that becomes more compact as the concentration of partially hydrolyzed polyacrylamide increases. The network structure of the weak gel system provides excellent shear resistance, with its apparent viscosity significantly higher than that of partially hydrolyzed polyacrylamide solution. The weak gel system exhibits typical pseudo-plastic behavior, which is a non-Newtonian fluid as well as a viscoelastic fluid. Additionally, the weak gel system’s elasticities exceed its viscosities, and longer crosslinking time further enhances the viscoelasticity. The weak gel system achieves superior conformance control and enhanced oil recovery in highly heterogeneous reservoirs compared to partially hydrolyzed polyacrylamide solutions. The weak gel system is more suited to low-permeability reservoirs with strong heterogeneity, as its effectiveness in conformance control and oil recovery increases with greater reservoir heterogeneity. Enhanced oil recoveries of the weak gel system in low-permeability sandpacks increase from 22% to 48% with a rise in permeability ratios from 14.39 to 35.64 after polymer flooding.

## 1. Introduction

During oil exploitation, extracting oil using the natural energy of the oil reservoir is typically referred to as primary oil recovery, achieving a recovery factor of only about 10% to 15% [1]. Extracting oil by injecting gas or water into the reservoir to maintain formation pressure is known as secondary oil recovery, which generally increases the recovery factor to around 30% to 40% [2]. Building on secondary oil recovery, tertiary oil recovery involves injecting chemical substances, gases, or microorganisms into the reservoir to alter the physical and chemical properties of the reservoir fluids, thereby further enhancing oil recovery [3,4,5,6]. Currently, the most widely used tertiary oil recovery methods in major oilfields domestically and internationally include chemical flooding, thermal flooding, gas miscible flooding, and microbial flooding [7,8,9,10]. In the past, the development of chemical flooding was limited due to low oil prices and the high cost of chemical agents. However, as oil demand and prices have risen, researchers increasingly select more economical and cost-effective chemical flooding techniques to efficiently extract remaining oil from reservoirs [11]. Chemical flooding is now considered one of the most promising methods for recovering residual oil in reservoirs [12]. This method enhances oil recovery by adding polymers, surfactants, alkalis, and other chemicals to injection water to modify the interactions between the displacement fluid and reservoir fluid [13]. Chemical flooding includes polymer flooding, surfactant flooding, alkaline flooding, and alkali–surfactant–polymer (ASP) flooding, among others [14].

Polymer flooding is a well-established enhanced oil recovery (EOR) technology and remains the most widely applied and effective tertiary recovery method in China’s oilfields [15,16]. By adding a specific amount of high-molecular-weight polyacrylamide to the injection water, polymer flooding increases the viscosity of the injected fluid, thereby improving the oil-water mobility ratio [17]. Once injected into the reservoir, the polymer solution exhibits high viscosity, a residual resistance coefficient, and viscoelastic properties. Higher polymer viscosity results in a greater residual resistance coefficient, which reduces the mobility of the displacement fluid. Consequently, a lower mobility ratio between the displacing and displaced fluids enhances both macroscopic and microscopic sweep efficiency in the reservoir, leading to improved EOR outcomes from polymer flooding [18,19]. However, during prolonged polymer injection, limitations in polymer concentration, molecular weight, and coiling prevent it from accessing inaccessible pores and from effectively blocking ultra-high-permeability channels over extended periods [20]. In the extended waterflooding phase, the profile control effect of the polymer tends to be short-lived, making it prone to fingering phenomena within the reservoir. When water breakthrough reoccurs in production wells, the water cut can increase rapidly [21,22]. Due to polymer adsorption on the rock surface, some polymer remains in the pores, reducing permeability in certain reservoir sections. Following polymer flooding, dominant seepage channels become more pronounced, and intra- and inter-layer as well as planar conflicts intensify [23]. Consequently, complex remaining oil patterns emerge due to polymer retention, shifts in pressure field, and changes in oil saturation, further complicating the oil displacement mechanism [24,25]. In medium- and low-permeability layers, improving water absorption becomes increasingly challenging, and subsequent displacement agents struggle to mobilize remaining oil effectively. Therefore, it is essential to develop new, safe, and efficient EOR technologies for mature oilfields following polymer flooding.

Currently, to further recover remaining oil in reservoirs after polymer flooding, extensive testing has been conducted on various technologies, including deep profile control, plug removal, combined chemical flooding, foam combination flooding, and microbial drive [26,27,28]. In particular, to reduce interlayer conflicts in reservoirs post-polymer flooding, various types of deep profile control agents have been developed [29,30]. These agents selectively block high-permeability channels upon injection, facilitating subsequent fluid diversion and thereby increasing the swept volume. Among these, polymer gel has been highly valued by petroleum science and technology workers due to significantly improve the fluid entry profile of water injection wells. Polymer gel refers to the system obtained by cross-linking reaction after adding cross-linking agent (such as multivalent metal ions or phenolic resin, etc.) into polymer solution (mainly polyacrylamide). The polymer gel used for deep profile control agent in oil field can be roughly divided into weak gel and colloidal dispersion gel [31]. The weak gel is a semifluid that is formed when linear polymers first undergo intramolecular cross-linking to create linked polymer coils, and subsequently, intermolecular cross-linking occurs between these linked polymer coils. The weak gel behaves as a pseudo-plastic fluid, with elasticity as its primary characteristic. A three-dimensional network structure forms via intra- and intermolecular cross-linking within the weak gel. In contrast, the colloidal dispersion gel represents a dispersion system consisting of spherical linked polymer coils within an aqueous solution that are formed through intramolecular cross-linking of linear polymers. Compared with the weak gel, the colloidal dispersion gel lacks a three-dimensional network structure, has a longer gelation time, and exhibits lower viscosity after gelation. Although effective for deep profile control, the colloidal dispersion gel is unsuitable for highly heterogeneous reservoir formations [32].

In reservoirs post-polymer flooding, weak gel can enter dominant channels to achieve effective plugging, adjusting permeability variations between oil layers to approximate a more homogeneous distribution, which facilitates piston-like displacement during subsequent waterflooding [33]. Due to prolonged water exposure, rock pore walls exhibit enhanced hydrophilicity and low friction against the water-based weak gel, leading to rapid gel seepage and creating a temporary negative pressure state within the pores [34]. This negative pressure allows the weak gel’s elasticity to overcome threshold pressure, converting bound crude oil into a free state and thereby forming an oil bank that moves forward. Additionally, the weak gel can modify the water injection profile by temporarily blocking high-permeability channels, enhancing local seepage resistance and diverting subsequent fluid flow to mobilize other oil layers, thus improving liquid absorption [35]. Under shear forces in porous media, large weak gel aggregates break into smaller clusters, enhancing overall gel fluidity, expanding the sweep range, and facilitating deep profile control after passing smoothly through the near-wellbore area [36]. During migration to deeper formations, these gel clusters reaggregate, blocking dominant channels in distal zones and altering the micro-stress distribution within the porous media [37]. Consequently, the gel clusters enable the recovery of remaining oil through the viscous forces exerted by the subsequent displacement fluid.

Due to the increasingly complex distribution of remaining oil and the intensifying interlayer and intralayer conflicts in reservoirs after polymer flooding, achieving a substantial increase in oil recovery has become challenging. In recent years, numerous studies have focused on gel profile control agents to further enhance oil recovery post-polymer flooding. Cao et al. developed a weak gel formulation system specifically for the Y1 block in the Daqing Yushulin Oilfield and evaluated the factors influencing weak gel formation through laboratory displacement experiments [38]. They determined that the optimal formulation of weak gel system contained 0.22% of polyacrylamide and 0.15% of chromium acetate system, with bacteria in the prepared water degrading the gel system, and certain insoluble suspended solids significantly improving its viscosity. Di et al. studied the oil displacement effects of various polymer-weak gel combinations (three combination patterns: waterflooding and polymer flooding; waterflooding, polymer flooding and weak gel flooding; waterflooding, weak gel flooding, and polymer flooding) by integrating nuclear magnetic resonance (NMR) imaging technology with core displacement experiments [39]. Their results showed that the combination of waterflooding, polymer flooding, and weak gel flooding provided the best profile control effect, achieving a maximum enhanced oil recovery of 18.33% based on the displacement efficiency of waterflooding. Zhang et al. investigated the dynamic sweep characteristics of well pattern densification, adjustment, and heterogeneous phase composite (HPC) flooding (using branched-preformed particle gel, which is a preformed particle gel with some branched chains, obtained by cross-linking acrylamide monomers, crosslinking agents, and other additives under certain conditions) after polymer flooding, along with their synergistic effects [40]. They concluded that well pattern densification and adjustment positively impacted HPC flooding, effectively expanding the swept volume due to the residual polymer’s synergistic effect. Despite these findings, weak gel systems are still rarely applied in reservoirs following polymer flooding, and evaluations of their displacement effects are limited. Given the complex characteristics of oil reservoirs after polymer flooding, further research is needed to assess the conformance control and enhanced oil recovery potential of weak gel systems in heterogeneous reservoirs.

In this study, we synthesized a weak gel system through the cross-linking reaction of chromium acetate and partially hydrolyzed polyacrylamide. Scanning electron microscopy (SEM) and Fourier-transform infrared (FT-IR) spectroscopy were used to examine the morphology and chemical structure of the weak gel system. We investigated the system’s viscous and viscoelastic properties under steady-shear conditions using a rheometer and analyzed how crosslinking time affected these properties. A series of parallel sandpack flooding experiments were conducted to assess the conformance control and enhanced oil recovery capabilities of the weak gel system in heterogeneous reservoirs following polymer flooding. Additionally, we examined the influence of permeability ratio on pressure drop, fractional flow, and enhanced oil recovery across waterflooding, polymer flooding, and weak gel flooding. These findings provide theoretical guidance for the extensive and effective development of similar reservoirs post-polymer flooding.

## 2. Results and Discussion

### 2.1. Morphology and Microstructure Characterization

**Figure 1:**
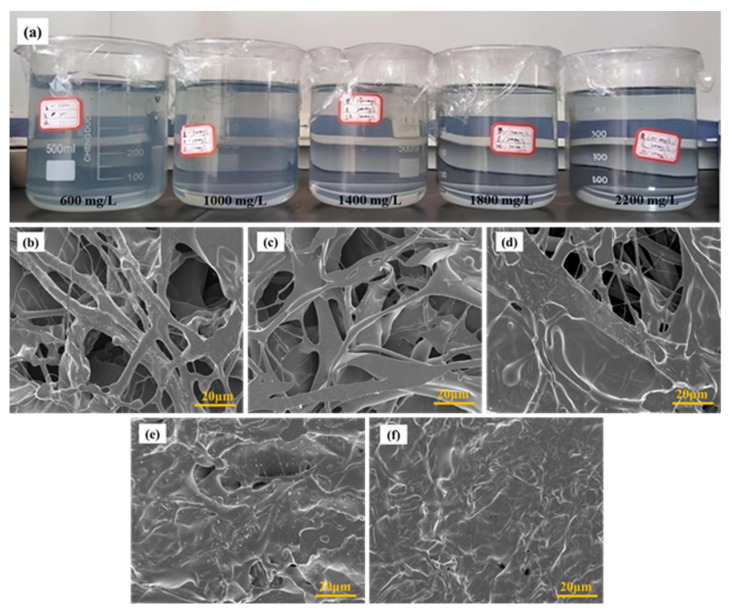
Physical photo (**a**) and SEM images of weak gels synthesized by different mass concentrations of partially hydrolyzed polyacrylamide: (**b**) 600 mg/L; (**c**) 1000 mg/L; (**d**) 1400 mg/L; (**e**) 1800 mg/L; (**f**) 2200 mg/L.

To investigate the influence of partially hydrolyzed polyacrylamide mass concentrations on the microstructures of weak gels, SEM measurements were conducted to characterize the morphologies of weak gels synthesized with varying concentrations of partially hydrolyzed polyacrylamide, as shown in Figure 1.

Figure 1a shows a physical photograph of the weak gels. According to the visual inspection, it can be observed that the solution becomes clearer as the concentration increases. As seen in Figure 1b–f, numerous polymeric microfibers are entangled to form network structures within the weak gels. The mass concentration of partially hydrolyzed polyacrylamide influences the overall spatial arrangement of these network structures. As the mass concentration of partially hydrolyzed polyacrylamide increases, the network structures in the weak gels become more compact. When the mass concentration is below 1400 mg/L, the main stems and branches within the network structures are more loosely distributed, with numerous pores present. The size of these pores gradually decreases with increasing mass concentration. Layered structures are observable within the weak gels, where each layer exhibits an arborization network connected by filamentous structures either within or between layers. When the mass concentration of partially hydrolyzed polyacrylamide exceeds 1400 mg/L, prominent white trunks and large black structural units, surrounded by these trunks, appear within the weak gels. These black structural units contain almost no pores, and as the mass fraction of partially hydrolyzed polyacrylamide increases, these units become even denser.

**Figure 2:**
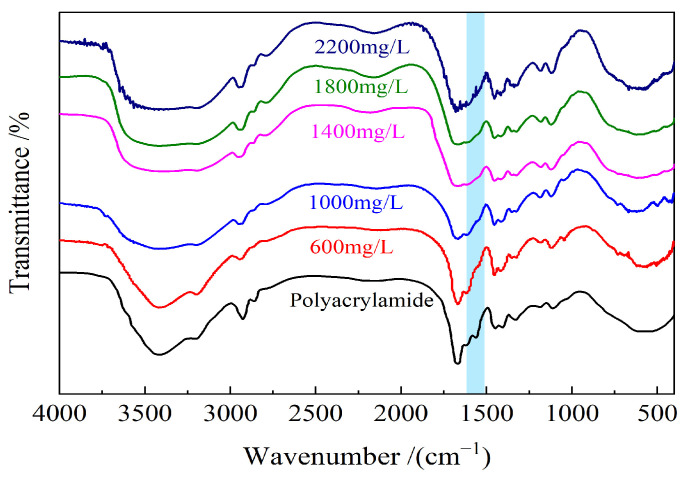
FT-IR spectra of partially hydrolyzed polyacrylamide and weak gels synthesized by different mass concentrations of partially hydrolyzed polyacrylamide.

The FT-IR spectra of partially hydrolyzed polyacrylamide and weak gels synthesized with different mass concentrations of partially hydrolyzed polyacrylamide are shown in Figure 2. The absorption peaks at 3420 and 3200 cm^−1^ are characteristic of free and associated amino groups, respectively [41]. The absorption peaks at 2920 and 2854 cm^−1^ correspond to the antisymmetric and symmetric stretching vibrations of the methylene group, respectively [41]. The peak at 1665 cm^−1^ is attributed to the stretching vibration of the C=O bond in the carbonyl group of the amide [42]. The peak at 1620 cm^−1^ is associated with the bending vibration of the N-H bond in the amide group, while the peak at 1452 cm^−1^ represents the deformation characteristic of the methylene group [42]. The peaks at 1180 and 1120 cm^−1^ are attributed to the stretching vibration of the C-N bond [43]. In comparison to the FT-IR spectrum of partially hydrolyzed polyacrylamide, the disappearance of the peak at 1560 cm^−1^, associated with the N-H bending vibration in the amide group, indicates the successful formation of the weak gel with a cross-linked network structure.

### 2.2. Rheological Properties of Weak Gel System

**Figure 3:**
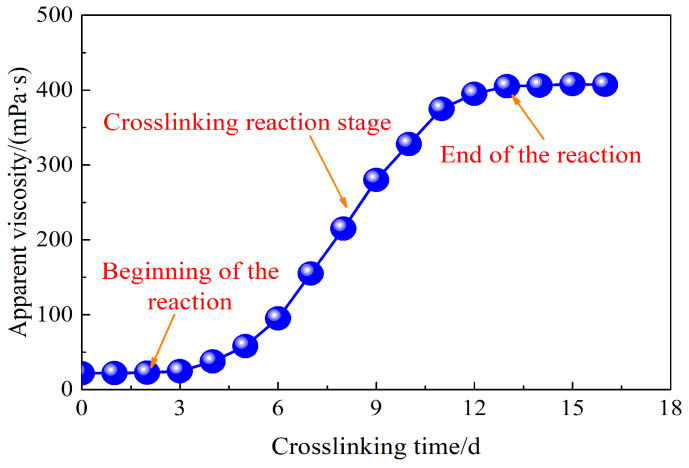
Effect of crosslinking time on the apparent viscosity of weak gel system crosslinked by the partially hydrolyzed polyacrylamide with a mass concentration of 1000 mg/L.

The weak gel system crosslinked by partially hydrolyzed polyacrylamide with a mass concentration of 1000 mg/L was prepared to determine the influence of crosslinking time on its rheological properties. Figure 3 shows the apparent viscosity of the weak gel system as a function of crosslinking time. As illustrated in Figure 3, the change in apparent viscosity of the weak gel system with crosslinking time can be divided into three stages. In the first stage, as the crosslinking reaction has not yet begun, the measured viscosity of the weak gel system essentially reflects the viscosity of the partially hydrolyzed polyacrylamide solution, showing little change as crosslinking time increases. In the second stage, once the crosslinking reaction initiates, the apparent viscosity of the weak gel system increases with crosslinking time. This phase demonstrates that the apparent viscosity of the weak gel system is time-dependent. In the third stage, as the crosslinking reaction completes, the apparent viscosity of the weak gel system stabilizes.

**Figure 4:**
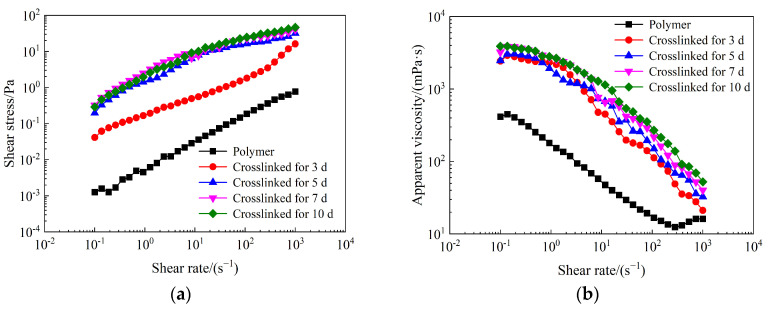
Effects of crosslinking time on rheological properties of weak gel system crosslinked by the partially hydrolyzed polyacrylamide with a mass concentration of 1000 mg/L: (**a**) shear stress; (**b**) apparent viscosity.

The rheological properties of weak gel systems at different crosslinking times were measured and compared with those of the partially hydrolyzed polyacrylamide solution. Figure 4a shows the shear stresses of weak gel systems as a function of shear rate across various crosslinking times. For weak gel systems crosslinked for 5, 7, and 10 days, the shear stress increases rapidly with the shear rate within the range of 0.1 to 100 s^−1^. Although the shear stress continues to rise as the shear rate increases from 100 to 1000 s^−1^, the rate of increase gradually declines. The shear stress–shear rate curve of the weak gel system crosslinked for 3 days differs from those of systems crosslinked for 5, 7, and 10 days, resembling more closely that of the polymer solution, as only a small amount of weak gel is formed after 3 days of crosslinking. Additionally, the shear stress of the weak gel system increases with extended crosslinking time, indicating that more gel structures are generated as crosslinking progresses. Furthermore, the shear stresses of weak gel systems at various crosslinking times are significantly higher than those of the partially hydrolyzed polyacrylamide solution.

Figure 4b shows the apparent viscosities of weak gel systems as a function of shear rate at different crosslinking times. The apparent viscosities of weak gel systems at various crosslinking times decrease as the shear rate increases from 0.1 to 1000 s^−1^. The weak gel systems exhibit shear-thinning behavior across different crosslinking times, indicating their typical pseudo-plastic properties [44], characteristic of non-Newtonian fluids within the shear rate range of 0.1–1000 s^−1^. Compared to the partially hydrolyzed polyacrylamide solution, the apparent viscosities of weak gel systems at various crosslinking times are significantly higher at the same shear rate. This increase in apparent viscosity may be attributed to the formation of high-molecular-weight gel structures, which result in greater viscosity once the crosslinking reaction initiates in the weak gel system [45].

The viscoelastic properties of weak gel systems at various crosslinking times were determined and compared with those of the partially hydrolyzed polyacrylamide solution. Viscoelasticity was characterized by measuring the complex modulus, storage modulus (elastic modulus), and loss modulus (viscous modulus) of both the partially hydrolyzed polyacrylamide solution and the weak gel systems at different crosslinking times. The term “complex modulus “ is introduced and defined as follows [45]:

(1) $$\left|G^{*}\right|=\sigma_{0}/\gamma_{0}$$

where G^*^ is the complex modulus that represents the energy of a material to resist deformation, Pa; *σ*_0_ is the amplitude of shear stress, Pa; *γ*_0_ is the amplitude of shear strain. The complex modulus can be expressed as follows [45]:

(2) $$G^{*}=G^{\prime }+iG^{\prime \prime }=\sigma_{0}\left(t\right)/\gamma_{0}\left(t\right)$$

Inside:

(3) $$G^{\prime }=G^{*}\cos\delta =\left(\sigma_{0}/\gamma_{0}\right)\cdot \cos\delta$$

(4) $$G^{\prime \prime }=G^{*}\sin\delta =\left(\sigma_{0}/\gamma_{0}\right)\cdot \sin\delta$$

(5) $$\tan\delta =\frac{{G^{\prime }}^{\prime }}{G^{\prime }}$$

where *G*′ is the storage or elastic modulus that represents the energy temporarily stored due to the reversible elastic deformation of a material, Pa; *G*″ is the loss or viscous modulus that represents the energy lost due to the irreversible viscous deformation of a material, Pa; *δ* is the phase angle that represents the ratio of viscosity to the elasticity of a material. The larger the phase shift angle is, the stronger the viscosity of a material to compare with its elasticity is. The smaller the phase shift angle, the stronger the material’s elasticity relative to its viscosity. The parameter *δ* can characterize the properties of fluids. When *δ* is in the range of 0–10°, it represents a strong elastic fluid. For *δ* ranging from 10° to 20°, it is an elastic fluid. When *δ* is between 20° and 40°, it is a viscoelastic fluid. And if *δ* is greater than 40°, it is a viscous fluid.

**Figure 5:**
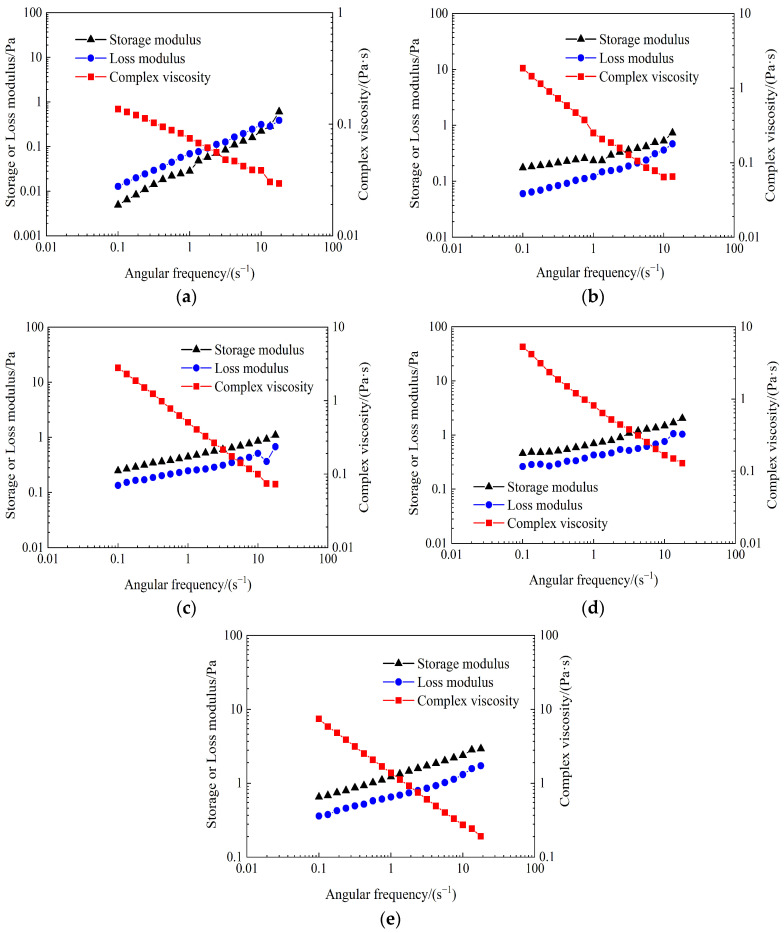
Viscoelastic curves of partially hydrolyzed polyacrylamide solution (**a**), and weak gel systems crosslinked for 3 (**b**), 5 (**c**), 7 (**d**), and 10 d (**e**) by the partially hydrolyzed polyacrylamide with a mass concentration of 1000 mg/L.

The frequency sweep measurements were conducted to determine η*, *G*′, and *G*″ of the partially hydrolyzed polyacrylamide solution and weak gel systems at various crosslinking times. The viscoelastic curves of the partially hydrolyzed polyacrylamide solution and weak gel systems at different crosslinking times are shown in Figure 5. As illustrated in Figure 5a, there is an intersection between the storage modulus and loss modulus curves of the partially hydrolyzed polyacrylamide solution as a function of angular frequency. The intersection occurs at an angular frequency of approximately 13.3 s^−1^. When the angular frequency is below 13.3 s^−1^, the loss modulus of the partially hydrolyzed polyacrylamide solution exceeds the storage modulus. Conversely, when the angular frequency is above 13.3 s^−1^, the storage modulus becomes slightly greater than the loss modulus. These results indicate that the partially hydrolyzed polyacrylamide solution exhibits stronger viscosity at low frequencies and stronger elasticity at high frequencies. As shown in Figure 5b–e, the storage moduli of the weak gel systems crosslinked for 3, 5, 7, and 10 days consistently remain higher than their loss moduli across the tested frequency range, indicating that the elastic properties of these weak gel systems dominate over their viscous properties at both low and high frequencies. A high storage modulus in a weak gel system is advantageous for enhancing the sweep efficiency of water flooding in heterogeneous reservoirs. This is because the weak gel system can create blockages in larger pore paths and pass through pore throats by deformation during displacement [46]. Therefore, compared to the partially hydrolyzed polyacrylamide solution, the weak gel system is expected to have a more beneficial effect on improving the sweep efficiency of water flooding.

**Figure 6:**
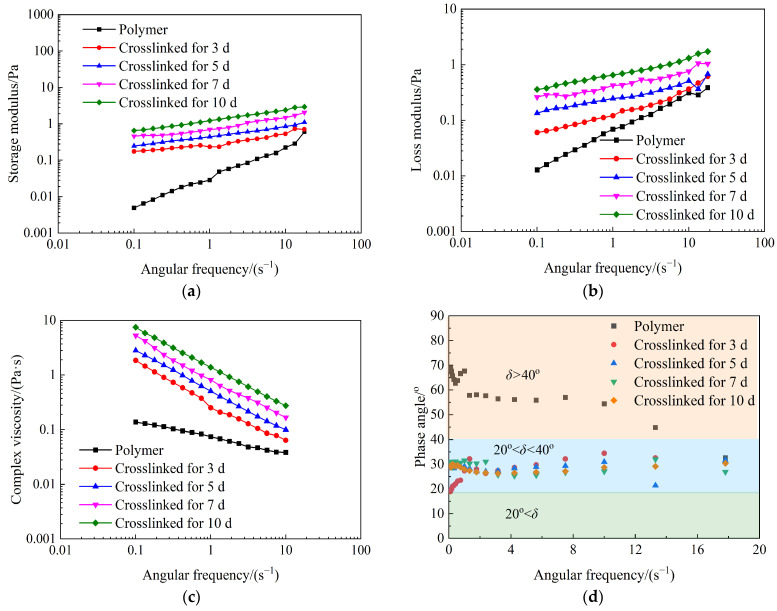
Effect of crosslinking time on the storage modulus (**a**), the loss modulus (**b**), the complex viscosity (**c**), and the phase angle (**d**) of weak gel systems crosslinked by the partially hydrolyzed polyacrylamide with a mass concentration of 1000 mg/L.

Figure 6 shows the storage modulus, loss modulus, complex viscosity, and phase angle of weak gel systems as functions of angular frequency at different crosslinking times. The storage moduli, loss moduli, and complex viscosities of weak gel systems all increase with extended crosslinking time. Compared to the partially hydrolyzed polyacrylamide solution, the storage modulus, loss moduli, and complex viscosities of weak gel systems are higher. As shown in Figure 6d, when the angular frequency is less than 13.3 s^−1^, the partially hydrolyzed polyacrylamide is a viscous fluid. Conversely, when the angular frequency is greater than 13.3 s^−1^, the partially hydrolyzed polyacrylamide displays characteristics of a viscoelastic fluid. Moreover, weak gels with different crosslinking times are all viscoelastic fluids. These results clearly indicate that the viscoelasticity of the weak gel system is stronger than that of the partially hydrolyzed polyacrylamide solution, and the viscoelasticity of the weak gel system strengthens with longer crosslinking times. Therefore, the weak gel system is more suitable than the partially hydrolyzed polyacrylamide solution for enhancing the sweep efficiency of water flooding in heterogeneous reservoirs.

### 2.3. Enhanced Oil Recovery in Parallel Sandpack Models

Three parallel sandpack flooding experiments were conducted to investigate the efficiency of the weak gel system in enhancing oil recovery in heterogeneous reservoirs after polymer flooding. In all three experiments, the weak gel system was prepared via the cross-linking reaction of partially hydrolyzed polyacrylamide with a mass concentration of 1000 mg/L. The permeabilities of the sandpacks and oil recoveries at different stages of the experiments are presented in Table 1. In tests 1, 2, and 3, the permeability ratios of the two sandpacks were 35.64, 21.47, and 14.39, respectively, with 0.2 PV (pore volume) of polymer solution and 0.2 PV of the weak gel system injected.

#### 2.3.1. Pressure Drop

**Figure 7:**
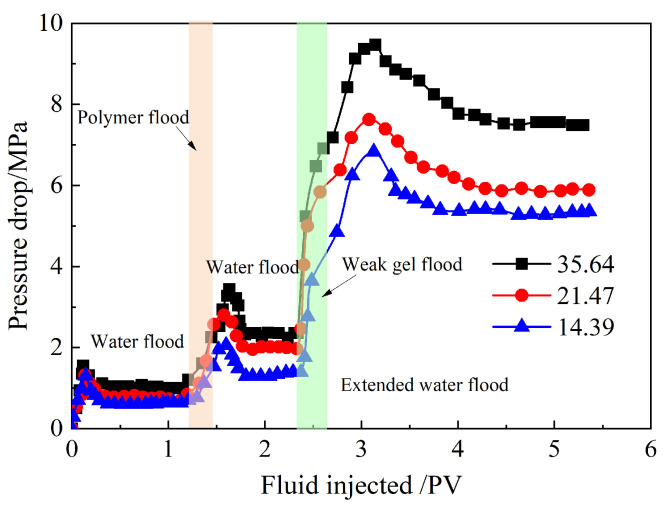
Pressure drop curves of three parallel sandpack flooding experiments with different permeability ratios.

**Figure 8:**
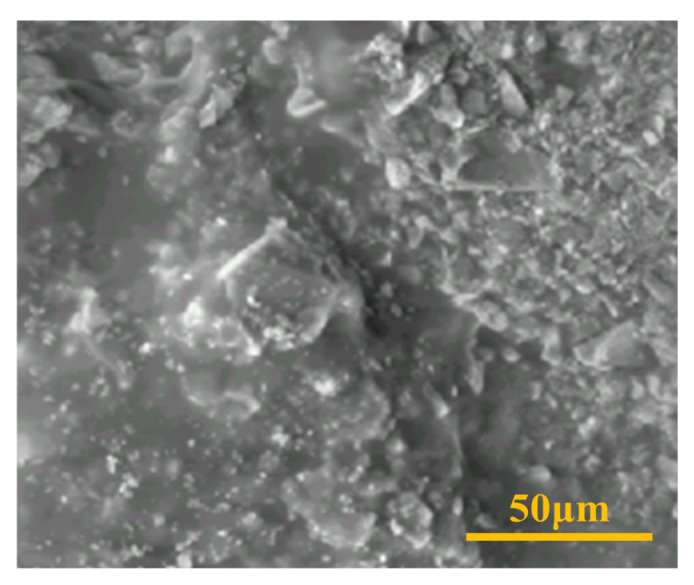
SEM image of the weak gel at the outlet of the sandpack with high permeability after flooding experiment was completed.

The pressure drop curves from the three parallel sandpack flooding experiments are shown in Figure 7. As seen in Figure 7, the variation of pressure drop with injected pore volume follows a similar trend in each experiment. The pressure drop increases rapidly with the initial injection of formation water into the sandpacks. After formation water breaks through at the outlet of the high-permeability sandpack, the pressure drop remains almost constant. With the injection of the partially hydrolyzed polyacrylamide solution, the pressure drop sharply rises, continuing to increase and reaching a maximum value during the extended waterflood. It then declines and stabilizes after the partially hydrolyzed polyacrylamide solution breaks through at the outlet of the high-permeability sandpack. Compared to the partially hydrolyzed polyacrylamide solution, the pressure drop rises at a faster rate when the weak gel is injected, reaching a higher maximum value during the extended waterflood. After the breakthrough of the weak gel system at the high-permeability sandpack outlet, the pressure drop decreases but remains at a higher level, indicating that the weak gel system provides stronger conformance control in heterogeneous reservoirs than the partially hydrolyzed polyacrylamide solution. The breakthrough of the weak gel system is confirmed by its SEM image at the sandpack outlet, as shown in Figure 8.

Additionally, Figure 7 shows that the permeability ratio has a significant impact on the pressure drop. The pressure drop across different parallel sandpacks increases with a higher permeability ratio. During the subsequent waterflood following the injection of the weak gel system, the maximum pressure drop rises from 6.83 MPa to 9.47 MPa as the permeability ratio increases from 14.39 to 35.64. These results indicate that the conformance control effect of the weak gel system strengthens with an increasing permeability ratio of the parallel sandpacks. The stronger the conformance control of the weak gel system in the high-permeability sandpack, the more subsequent water flow is directed into the low-permeability sandpack. Consequently, more crude oil is displaced from the sandpacks, leading to a higher enhanced oil recovery (EOR). Therefore, it can be concluded that the weak gel system exhibits greater conformance control in reservoirs with higher heterogeneity.

#### 2.3.2. Fractional Flows

**Figure 9:**
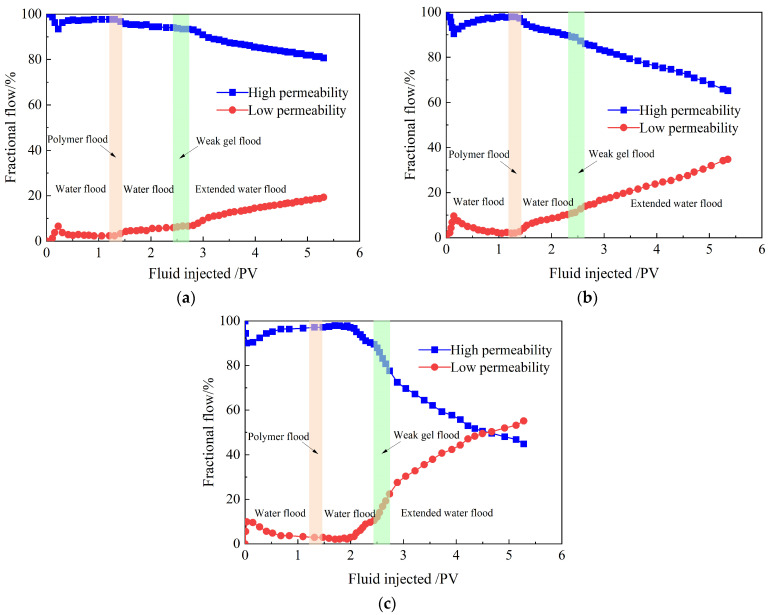
Fractional flow curves of three parallel sandpack flooding experiments. Permeability ratio: (**a**) 14.39; (**b**) 21.47; (**c**) 35.64.

The fractional flow curves for the five flooding stages—comprising the initial waterflood, partially hydrolyzed polyacrylamide slug, second waterflood, weak gel slug, and extended waterflood—in three parallel sandpack flooding experiments are shown in Figure 9. In all three experiments with different permeability ratios, the fractional flow curves exhibit similar trends across the five flooding stages. During the initial waterflood stage, the difference in fractional flow between the high- and low-permeability sandpacks increases as formation water is injected. When formation water breaks through in the high-permeability sandpack, the low-permeability sandpack is barely affected by the waterflood. As illustrated by the fractional flow curves in Figure 9, the fractional flow exceeds 90% in the high-permeability sandpack within the range of 0.5 to 1.2 PV and is less than 10% in the low-permeability sandpack. When 0.2 PV of partially hydrolyzed polyacrylamide solution is injected and the second waterflood begins, the fractional flow in the low-permeability sandpack increases while it decreases in the high-permeability sandpack. By the end of the second waterflood, the fractional flow exceeds 10% in the low-permeability sandpack and drops below 90% in the high-permeability sandpack, indicating that the partially hydrolyzed polyacrylamide solution provides only limited conformance control. As 0.2 PV of the weak gel system is injected and the extended waterflood commences, the fractional flow in the low-permeability sandpack rises rapidly, while it decreases in the high-permeability sandpack. At the conclusion of the extended waterflood, the fractional flow reaches over 20% in the low-permeability sandpack and falls below 80% in the high-permeability sandpack. This indicates that most of the weak gel enters the high-permeability sandpack, effectively blocking water flow channels there and diverting subsequent water flow toward the low-permeability sandpack.

Moreover, Figure 9 shows that the permeability ratio has a limited effect on fractional flow during the initial waterflood, partially hydrolyzed polyacrylamide slug, and second waterflood stages in the three parallel sandpack flooding experiments. However, the permeability ratio significantly influences fractional flow during the weak gel slug and extended waterflood stages. At the end of the initial waterflood, the differences in fractional flows show minimal change across the three experiments, remaining above 95%. This indicates that waterflooding is less effective in reservoirs with strong heterogeneity. At the end of the second waterflood, the difference in fractional flows between the two sandpacks decreases from 88% to 52% as the permeability ratio increases from 14.39 to 35.64, indicating that the conformance control effect of the partially hydrolyzed polyacrylamide solution strengthens as reservoir heterogeneity increases. By the conclusion of the extended waterflood, the difference in fractional flows decreases from 61% to 30% as the permeability ratio increases from 14.39 to 21.47. Notably, the fractional flow in the low-permeability sandpack exceeds that in the high-permeability sandpack when the permeability ratio reaches 35.64. This reversal in fractional flow demonstrates that the more heterogeneous the reservoir, the more effective the conformance control of the weak gel system. When the weak gel system enters the sandpacks, it effectively blocks the main water flow channels in the high-permeability sandpack, increases flow resistance in the blocked regions, and redirects the subsequent water flow toward the low-permeability sandpack. As a result, the weak gel system effectively reduces reservoir heterogeneity and expands the swept volume of the following water flow.

#### 2.3.3. Enhanced Oil Recovery

**Figure 10:**
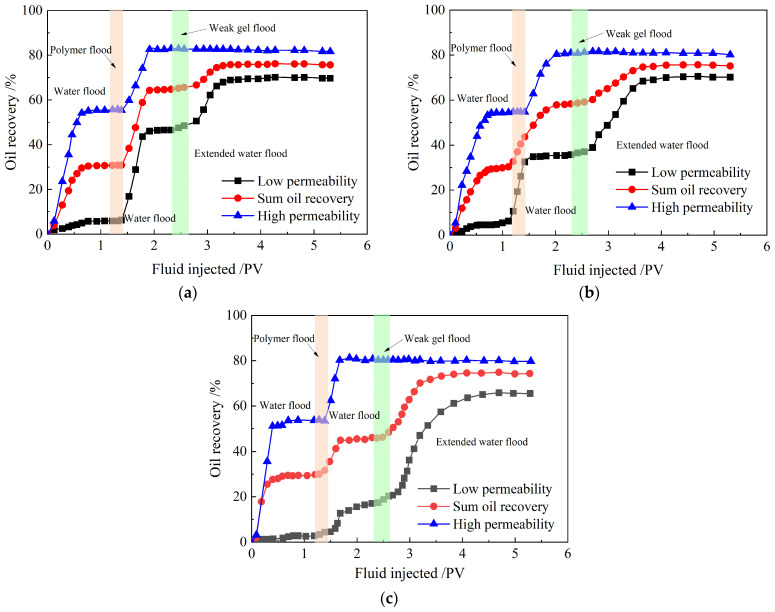
Cumulative oil recovery curves of three parallel sandpack flooding experiments. Permeability ratio: (**a**) 14.39; (**b**) 21.47; (**c**) 35.64.

The cumulative oil recovery curves from the three parallel sandpack flooding experiments are shown in Figure 10. Each diagram in Figure 10 includes three curves representing the high-permeability sandpack, the low-permeability sandpack, and the total oil recovery, respectively. The cumulative oil recovery encompasses all five flooding stages: initial waterflood, partially hydrolyzed polyacrylamide slug, second waterflood, weak gel slug, and extended waterflood. The results from the three experiments indicate that oil recovery in the high-permeability sandpack is significantly higher than that in the low-permeability sandpack during the initial waterflood stage. At permeability ratios of 14.39, 21.47, and 35.64, the initial waterflood recovers 56%, 55%, and 54% of the initial oil in place (IOIP) in the high-permeability sandpacks, compared with only 6%, 11%, and 3% IOIP in the low-permeability sandpacks. This indicates that a large amount of residual oil remains in the low-permeability sandpack. From the trend of the oil recovery curves in the initial waterflood stage, it is evident that the oil recovery in the low-permeability sandpack cannot match that of the high-permeability sandpack through waterflooding alone.

To improve the sweep efficiency of the heterogeneous sandpacks, 0.2 PV of partially hydrolyzed polyacrylamide solution was injected, followed by a 1.0 PV second waterflood, aimed at enhancing oil recovery in both the low- and high-permeability sandpacks. During this stage, the oil recovery curves from the three parallel flooding experiments indicate that oil recoveries in both the low- and high-permeability sandpacks increase. When the permeability ratio is 14.39, oil recovery in the low-permeability sandpack increases more rapidly than in the high-permeability sandpack. However, as the permeability ratio rises to 35.64, oil recovery in the low-permeability sandpack increases at a slower rate compared to the high-permeability sandpack. This suggests that the conformance control effect of the partially hydrolyzed polyacrylamide solution is more effective in reservoirs with weaker heterogeneity than in those with stronger heterogeneity. At permeability ratios of 14.39, 21.47, and 35.64, after the second waterflood, oil recoveries in the high-permeability sandpacks reach 83%, 81%, and 80% IOIP, respectively, which are still considerably higher than those in the low-permeability sandpacks—48%, 36%, and 17% IOIP, respectively. This illustrates that a significant amount of residual oil remains in the low-permeability sandpacks after the application of partially hydrolyzed polyacrylamide solution for conformance control, with higher permeability ratios leading to more residual oil.

To further enhance oil recovery in the low-permeability sandpacks, 0.2 PV of the weak gel system was injected, followed by a 2.8 PV extended waterflood. The oil recovery curves from the three parallel flooding experiments during this stage indicate that oil recovery in the high-permeability sandpacks remains nearly constant, meaning no additional crude oil is displaced from these sandpacks. However, oil recovery in the low-permeability sandpacks increases rapidly, and the higher the permeability ratio, the faster the increase in oil recovery. At permeability ratios of 14.39, 21.47, and 35.64, the oil recoveries in the low-permeability sandpacks after the extended waterflood reach 70%, 70%, and 66% IOIP, respectively, which are close to but still slightly lower than those of the high-permeability sandpacks. This occurs because most of the injected weak gel system enters and remains in the high-permeability sandpacks. The retained weak gel system exerts a sustained effect on sweep efficiency during the extended waterflood following the injection of the weak gel slug, diverting subsequent water flow to the low-permeability sandpacks. These results demonstrate that the weak gel system provides better conformance control in strongly heterogeneous reservoirs compared to the partially hydrolyzed polyacrylamide solution.

The results in the third-to-last column of Table 1 show that the enhanced oil recoveries in the high-permeability sandpacks due to the partially hydrolyzed polyacrylamide solution slug and second waterflood are 27%, 26%, and 26%, respectively, while those in the low-permeability sandpacks are 42%, 26%, and 14%, respectively, at permeability ratios of 14.39, 21.47, and 35.64. These findings indicate that injecting a partially hydrolyzed polyacrylamide solution slug effectively improves sweep efficiency and recovers some residual oil in low-permeability sandpacks. However, the sweep efficiency of the partially hydrolyzed polyacrylamide solution decreases as the permeability ratio increases, suggesting that it is more suitable for improving sweep efficiency in low-permeability reservoirs with weak heterogeneity. The results in the last column of Table 1 indicate that the enhanced oil recoveries in the high-permeability sandpacks are all 0% at permeability ratios of 14.39, 21.47, and 35.64 following the injection of the weak gel system slug and extended waterflood. In contrast, the oil recoveries in the low-permeability sandpacks are 22%, 34%, and 48%, respectively. As is evident from Table 2, in contrast to the currently available partial flooding systems, the weak gel system demonstrates a remarkable tertiary oil displacement efficacy. These results demonstrate that the weak gel system slug more effectively improves sweep efficiency and recovers a greater amount of residual oil in low-permeability sandpacks compared to the partially hydrolyzed polyacrylamide solution. Furthermore, the sweep efficiency of the weak gel system strengthens with increasing permeability ratio, implying that it is better suited for enhancing sweep efficiency in low-permeability reservoirs with strong heterogeneity. Therefore, in more heterogeneous reservoirs, the effect of the weak gel system on sweep efficiency becomes more pronounced following partially hydrolyzed polyacrylamide solution flooding.

## 3. Conclusions

The weak gel systems were synthesized through the cross-linking reaction of chromium acetate and partially hydrolyzed polyacrylamide with different mass concentrations. SEM measurements and Fourier-transform infrared (FT-IR) spectroscopy confirmed their morphologies and chemical structures. The viscous and viscoelastic properties of the weak gel systems at various crosslinking times, ranging from 3 to 10 days, were investigated under steady-shear conditions. The conformance control and enhanced oil recovery capabilities of the weak gel system after polymer flooding in a heterogeneous reservoir were assessed using parallel sandpack flooding experiments. The following conclusions are summarized:

(1) The synthesized weak gel system flaunts a distinct network structure, endowing it with excellent shear resistance. Its apparent viscosity is notably higher than that of the partially hydrolyzed polyacrylamide solution and rises with prolonged crosslinking time. Moreover, it exhibits typical pseudo-plastic behavior.
(2) The weak gel system belongs to the category of viscoelastic fluids, wherein its elastic properties prevail over the viscous ones. The weak gel system exhibits notably enhanced viscoelastic properties. As the crosslinking process extends over a longer duration, the viscoelasticity of the weak gel system experiences a progressive augmentation, manifesting a more pronounced and resilient rheological behavior.
(3) Compared with the partially hydrolyzed polyacrylamide solution, the weak gel system is better suited for highly heterogeneous, low-permeability reservoirs. The more heterogeneous the reservoir, the more effective the weak gel’s conformance control. After partially hydrolyzed polyacrylamide solution flooding, as the permeability ratio climbs from 14.39 to 35.64, enhanced oil recoveries in low-permeability sandpacks soar from 22% to 48%.

## 4. Materials and Methods

### 4.1. Materials

Chromium acetate, thiourea, and partially hydrolyzed polyacrylamide (with a relative molecular weight of 12 million and a hydrolysis degree of 20%) were purchased from Macklin Biochemical Co., Ltd. (Shanghai, China) for the preparation of the weak gel. Sodium sulfate, potassium chloride, magnesium chloride, sodium bicarbonate, and calcium chloride were obtained from Sinopharm Chemical Reagent Co., Ltd. (Beijing, China) to prepare simulated formation water. All reagents were of analytical grade, and solutions were prepared using deionized water. The synthetic formation water used in the flooding experiments was a brine with a total dissolved solids (TDS) concentration of 6805 mg/L, where the mass concentrations of K^+^, Na^+^, Ca^2+^, Mg^2+^, HCO_3_^−^, Cl^−^, and SO_4_^2−^ were 1215, 1215, 45, 45, 739, 3466, and 80 mg/L, respectively. The crude oil sample for the flooding experiments was sourced from a Shengli oil reservoir, with a density of 0.907 g/cm^3^ and a viscosity of 28.4 mPa·s at 50 °C.

### 4.2. Preparation of Weak Gel

**Figure 11:**
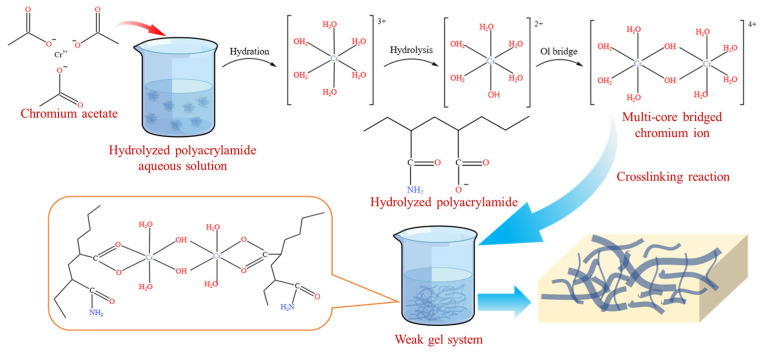
Schematic illustration of the synthesis of weak gel cross-linked from chromium acetate and partially hydrolyzed polyacrylamide.

A specified amount of partially hydrolyzed polyacrylamide was weighed and added to deionized water to prepare partially hydrolyzed polyacrylamide solutions with mass concentrations of 600, 1000, 1400, 1800, and 2200 mg/L. Chromium acetate (100 mg) was then slowly added as a crosslinking reagent, along with thiourea (50 mg) as a stabilizer, into the different partially hydrolyzed polyacrylamide solutions. The resulting solutions were continuously stirred for 10 min at 100 rpm and then placed in an incubator to form the weak gels via cross-linking at 50 °C for at least 3 days. A schematic illustration of the synthesis process for the weak gel cross-linked from chromium acetate and partially hydrolyzed polyacrylamide as shown in Figure 11.

### 4.3. Characterization

After completing the crosslinking reaction and successfully preparing the weak gels, a specific volume of liquid weak gel was taken from each solution and freeze-dried for 24 h to obtain solid weak gels using a vacuum freeze dryer (Tianling Instrument Co., Ltd., Yancheng, China). After the gold sputtering treated for block-shaped weak gels, their morphologies were examined using scanning electron microscopy (SEM, Sigma 500, Carl Zeiss, Oberkochen, Germany). The functional groups of the weak gels and partially hydrolyzed polyacrylamide were characterized via Fourier-transform infrared spectroscopy (FT-IR, 6700 Thermo Nicolet, Madison, WI, USA).

### 4.4. Rheological Experiment

The rheological measurements were conducted using a rheometer (MCR300 Paar Physica, Ostfildern, Germany) equipped with a coaxial cylinder sensor system. The cylinder has an internal diameter of 43.2 mm, and the gap between the inner and outer cylinders is 0.8 mm. The apparent viscosities of the weak gel crosslinked by partially hydrolyzed polyacrylamide with a mass concentration of 1000 mg/L were measured at a shear rate of 7.34 s^−1^ and at 50 °C to investigate the effect of crosslinking time. The shear stresses and apparent viscosities versus shear rates for the partially hydrolyzed polyacrylamide and the weak gels crosslinked for 3, 5, 7, and 10 days were determined at shear rates ranging from 0.1 to 1000 s^−1^ and at 50 °C. Subsequently, frequency sweep measurements were performed within a frequency range of 0.1–100 Hz at shear stress of 0.1 Pa to determine the complex viscosities, storage moduli, and loss moduli of the partially hydrolyzed polyacrylamide solution and the weak gels crosslinked for 3, 5, 7, and 10 days.

### 4.5. Parallel Sandpack Flooding Experiment

**Figure 12:**
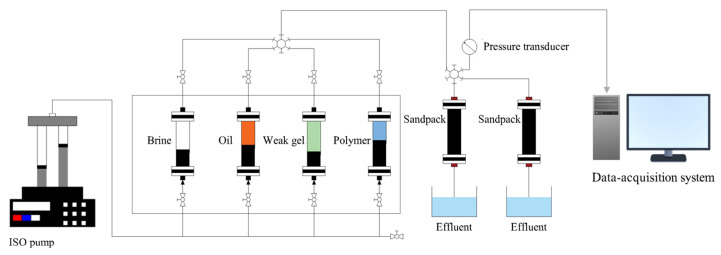
Schematic diagram of the parallel sandpack flooding experiment.

The sandpacks were used to conduct the parallel flooding experiments. Fresh quartz sands with a diameter of 30–40 μm were packed into stainless steel holders to prepare the sandpacks, which had a length of 40 cm and a diameter of 25 cm. Figure 12 shows a schematic diagram of the parallel sandpack flooding experiment. The experimental setup consisted of two sandpacks, a pressure transducer connected to a computer for continuous recording of the inlet pressure, a syringe pump for injecting fluids at a constant flow rate, and two measuring cylinders to collect the effluents from the outlets of the sandpacks. The porosity of the sandpacks was determined using the weight method, and their permeability was measured according to Darcy’s Law for single-phase water flow. All parallel sandpack flooding experiments were conducted at 50 °C. In the parallel sandpack flooding experiments, the sandpacks were initially saturated with formation brine and then displaced with crude oil at a flow rate of 0.2 mL/min until the initial oil saturations were established in both sandpacks. The sandpacks were aged for 48 h to achieve the desired wettability state at 50 °C. Following this, the sandpacks were flooded with formation brine (1.2 PV) at a flow rate of 0.2 mL/min. A slug (0.2 PV) of partially hydrolyzed polyacrylamide solution with a mass concentration of 1000 mg/L was injected at a flow rate of 0.2 mL/min, followed by a waterflood (1.0 PV). Finally, a slug (0.2 PV) of weak gel crosslinked by partially hydrolyzed polyacrylamide with a mass concentration of 1000 mg/L was injected at a flow rate of 0.5 mL/min, followed by an extended waterflood (2.8 PV) until the oil cut was less than 2%. Oil production and injection pressure data were continuously recorded throughout the flooding experiment.

## Figures and Tables

**Table 1:** Efficiency of cumulative oil recovery (COR) and enhanced oil recovery (EOR) in heterogeneous parallel sandpacks.

Test	Permeability (10^−3^ μm^2^)	Permeability Ratio	Initial oil Saturation (%)	Recovery of Water Flood (%)	After Polymer Flooding		After Weak Gel Flooding	
					COR (%)	EOR (%)	COR (%)	EOR (%)
1	High 4740.82	35.64	78	54	80	26	80	0
	Low 133.02		82	3	17	14	66	48
2	High 3278.17	21.47	78	55	81	26	81	0
	Low 152.69		84.	11	36	26	70	34
3	High 2633.68	14.39	77	56	83	27	83	0
	Low 183.02		88	6	48	42	70	22

**Table 2:** Effectiveness of the presently accessible partial flooding systems in relation to tertiary oil recovery.

Flooding System	Incremental Oil Recovery (%)	Ref
Weak gel flooding after polymer flooding	18	Di et al. [39]
ASP flooding after polymer flooding	19	Wang et al. [16]
Heterogeneous combination flooding after polymer flooding	25	Wang et al. [16]
HPC flooding after polymer flooding	14	Zhang et al. [40]

## Data Availability

The data that support the findings of this study are available from the corresponding authors upon reasonable request.
